# The Economic Burden of Infective Endocarditis due to Injection Drug Use in Australia: A Single Centre Study—University Hospital Geelong, Barwon Health, Victoria

**DOI:** 10.1155/2022/6484960

**Published:** 2022-12-16

**Authors:** Ohide Otome, Alexander Wright, Vanika Gunjaca, Steve Bowe, Eugene Athan

**Affiliations:** ^1^University of Western Australia, Perth, Australia; ^2^SJOG Midland Public and Private Hospital, Midland, Australia; ^3^University Hospital Geelong, Barwon Health, Geelong, Australia; ^4^Deakin Biostatistics Unit, Faculty of Health, School of Medicine, Deakin University, Geelong, Australia; ^5^Geelong Centre for Emerging Infectious Disease (GCEID), Geelong, Australia

## Abstract

**Background:**

Injection drug use (IDU) is a well-recognized risk factor for infective endocarditis (IE). Associated complications from IDU result in significant morbidity and mortality with substantial cost implications. The aim of this study was to determine the cost burden associated with the management of IE due to IDU (IE-IDU).

**Methods:**

We used data collected prospectively on patients with a diagnosis of IE-IDU as part of the international collaboration on endocarditis (ICE). The cost of medical treatment was estimated based on diagnosis-related groups (DRG) and weighted inlier equivalent separation (WIES).

**Results:**

There were 23 episodes from 21 patients in 12 years (2002 to 2014). The costing was done for 22 episodes due to data missing on 1 patient. The median age was 39 years. The gender distribution was equal. Heroin (71%) and methamphetamine (33%) were the most frequently used. 74% (17/23) required intensive care unit (ICU) admission. The median ICU length of stay (LOS) was 4 days (IQR (Interquartile range); 2 to 40 days) whilst median total hospital LOS was 40 days (IQR; 1 to 119 days). Twelve patients (52%) underwent valve replacement surgery. Mortality was 13% (3/23). The total medical cost for the 22 episodes is estimated at $1,628,359 Australian dollars (AUD). The median cost per episode was a median cost of $ 61363 AUD (IQR: $2806 to $266,357 AUD). We did not account for lost productivity and collateral costs attributed to concurrent morbidity.

**Conclusion:**

Within the limitations of this small retrospective study, we report that the management of infective endocarditis caused by injection drug use can be associated with significant financial cost.

## 1. Background

Injection drug use in Australia is a cause of considerable morbidity [1, 2]. It is one of the most common modes of illicit drug use, with a combined (methamphetamine, cocaine, heroin) prevalence of approximately 29%, behind alcohol (86%), tobacco (39%), and cannabis (35%) [3, 4]. IDUs (injection drug users) are more likely to be from low-socioeconomic communities with a predilection for drug use due to historical reasons, such as being part of a subculture [1, 2]. IDU in turn results in mental and physical health problems including but not limited to depression, aggression, anxiety disorders, and personality problems [5, 6]. Infection with blood borne viruses (hepatitis B, C, and HIV) and bacterial infection due to unsafe injection techniques make up one of the most common complications [7, 8].

At presentation, when IE is suspected, urgent hospitalization is necessary to allow for investigation and management. This often mandates a multidisciplinary specialist input and a number of tests and interventions [9]. Intensive care (ICU) admission and, in some cases, cardiac surgery are required. The length of stay (LOS) is prolonged due to a slow clinical recovery and complex discharge planning due to poor or a lack of the necessary social support networks [9]. The morbidity resulting from complications of IE can often be severe enough to disrupt an individual's capacity to participate in the workforce, having a deleterious effect on their economic productivity [10]. It is on this basis that we would like to understand the cost burden on the health care budget. The aim of our research was therefore to determine the cost of providing medical treatment to a cohort of patients with a history of IDU who presented with a diagnosis of definite IE.

## 2. Methods

### 2.1. Study Site

This study was undertaken at the University Hospital Geelong, a 406-bed regional hospital located in south-west Victoria. It is the only cardiothoracic centre in the region with a catchment population of over 500,000 as of 2014 [11]. As such, it is likely to see a significant number of presentations related to the complications of IDU.

### 2.2. Ascertainment and Data Collection

University hospital Geelong collects and contributes data to the International Collaboration on Endocarditis database [12]. This is an active prospective multicenter database aiming to pool data from a large cohort of IE patients to facilitate research in this area. The background and inclusion criteria have been previously reported [12]. We used this dataset to retrospectively collect data on demographics (age, sex, and ethnicity), clinical (vital signs, including radiological and blood test results) microbiological (including blood culture results), and echocardiographic results. This was limited to patients identified as IDUs based on a self-reported history of injection drug use within the last 3 months.

### 2.3. Ethical Approval

This study was approved by the Barwon Health institutional review board as part of the ongoing IE study.

### 2.4. Definitions

Weighted in-lier equivalent separation (WIES) is a cost “weight” (*W*) that is adjusted for time spent in hospital “in-lier equivalent separation” (IES) and represents a relative measure of resource use for each episode of care in a DRG (diagnosis-related group). WIES allocated to an episode depends on the episode of DRG, the amount of time spent in the hospital and the episodes' eligibility for WIES copayments. In the state of Victoria, WIES cost weights are developed each year using the costs of treating patients as reported to the department of health and human services by public hospitals. The department pays a price per unit of WIES. WIES prices vary between hospitals to account for differences in specialisation, economies of scale, and levels of remoteness. To determine the dollar value, the cost weight is multiplied by the dollar rate applicable for the financial year [13].

Diagnosis-related groups (DRG) are a patient categorisation system that regulates prospective payment to hospitals and promotes cost containment initiatives. In general, a DRG payment includes all charges related to an inpatient stay from the point of admission to discharge [13].  Table 1 represents episodes of presentations, DRG funding, and an estimation of cost based on WIES. There are a variety of DRGs in this cohort that influence funding for each episode. As an example, DRGs beginning with “A” identify very high-cost episode e.g., A06A tracheostomy >95 hours, which are driven by a specific intervention code that overrides the outcome of the principle diagnosis [14].

Infective endocarditis (IE) is an infection of the cardiac endothelium; it does typically involve the cardiac valves as well as indwelling cardiac devices [15].

Injection drug use (IDU) is the voluntary practice of administration of recreation drugs such as methamphetamine, cocaine, or heroin for the purpose of enjoying their stimulatory or sedative effects [5]. In the majority of cases, this is administered via the intravenous route, thereby increasing the risk of bacterial contamination if performed using non-sterile techniques. This in turn predisposes individuals to bacterial complications including skin and soft tissue infections at the injection site and, in severe cases, infective endocarditis [16].

### 2.5. Statistics

The data was recorded in Microsoft Excel and described as proportions, means (±SD) or median (range).

### 2.6. Costing

All patients were treated under the public hospital scheme. The government of the state of Victoria uses WIES to calculate cost and funding for hospitals based on a number of factors including DRG, admission type, LOS, hospital location, and performance of some procedures e.g., cardiac surgery [17–19]. Inflation was calculated using the consumer price index (CPI) [17–19] for each year and 2014 to determine costs as of 2014. The CPI for the relevant years was obtained from the Australian Taxation office [17, 19, 20]. The cost is expressed as AUD.

## 3. Results

We had 23 episodes from 21 patients over 12 years (2002 to 2014). Costing was only done for 22 episodes (DRG data for one patient in 2002 could not be retrieved) (Table 1). All patients were Australian born. The median age was 39 years. The gender distribution was equal (females 12 and males 11) (Table 2). Hepatitis C was the most prevalent comorbidity (76%). Hepatitis B and HIV were reported in one patient each. Psychiatric conditions (depression, anxiety, schizophrenia, and PTSD), smoking, and alcohol use ranked high on the list of associated conditions 52%, 36%, and 27%, respectively. Heroin and methamphetamine were the most frequently injected drugs, at 71.4% and 33.3%, respectively.

Of all the presentations, more than two-thirds (74%; (17/23)) required ICU admission. The median ICU LOS was 4 days (range 2–40 days). Of those in the ICU, 88% (15/17) required invasive ventilation, of which 4 had tracheotomies. Isolated RSIE (right side IE) was identified in 47% (11/23), LSIE (left side IE) in 34% (8/23), and left and right sided IE were seen in only 3 (13%) patients. Twelve patient episodes required valve surgery (52%, 12/23) (Table 2). All underwent valve replacement. Of those who underwent valve surgery 6 were RSIE all due to MSSA; 5 had LSIE (2 MSSA, 2 *Streptococcus viridans* group, and 1 *Candida albicans*). Bilateral left and right sided IE was documented in only one patient with MSSA who also needed cardiac surgery. The indications identified in all 12 were heart failure due to acute valve dysfunction and uncontrolled sepsis. Other infectious complications in order of frequency included septic pulmonary emboli 60% (14/23). This was seen in 100% of those with RSIE. Cerebral emboli was seen in 50% (4/8) of patients with LSIE. Discitis with vertebral osteomyelitis was seen in 3/23 (13%) patients and septic arthritis in 2/23 (8%) patients. The most common noninfectious complication was acute kidney injury (AKI) and all 6/23 (26%) received hemofiltration. One patient with AKI went on to require long term dialysis.

Medical imaging was used as follows: magnetic resonance imaging (MRI) 0.7 per episode (17 scans in 23 episodes), computed tomography 1.7 per episode (39 scans in 23 episodes), plain X-ray 9 per episode (214 scans in 23 episodes), ultrasound abdomen 0.7 per episode (17 scans in 23 episodes). *Staphylococcus aureus* was the most common infection, seen in 82% (19/23), followed by *Streptococcus species* 13% (3/23). There were two cases of MRSA (methicillin-resistant *Staphylococcus aureus*) IE and one case of *Candida* sp. fungal endocarditis. There were a total of two Candida blood stream infections (primary candidemia and secondary line-related infection) and one VRE blood stream infection attributed to central line-related sepsis in the ICU. All patients but one had antibiotics administered as inpatients. The average duration was 37 days.

Caring for IDU-IE is often a multidisciplinary team effort. There was evidence of multiple subspecialist team involvement, including but not limited to: infectious diseases, general medicine, cardiology, ICU, cardiothoracic surgery, vascular surgery, drug and alcohol abuse, orthopaedics, nephrology, general surgery, and plastic surgery. Some patients were referred onward for neurosurgical intervention at a secondary hospital in Melbourne, Victoria. The mean hospital LOS was 40 days. The majority (>70%) were discharged home and 3 were sent to other hospitals. Mortality was reported in 3 patients (13%). All deaths occurred <30 days of hospital admission. The cause of death in all was severe sepsis and multiorgan failure.

The total medical cost for the 22 episodes is estimated at $1,628,359 AUD with a calculated median cost of $61363 AUD (IQR $2806 to $266,357 AUD) (Table 1) [13].

## 4. Discussion

The economic cost resulting from complications of IDU is the cause of immense burden to society [21]. Although NSP (needle syringe exchange programs) have been found cost-effective, the main benefit has mostly been a reduction in the incidence of blood borne viruses (hepatitis C, hepatitis B, and HIV) and their associated complications [21]. Meanwhile, bacterial infections continue to cause significant morbidity and are responsible for the substantial mortality seen in this cohort [22]. In support of this, Tung et al. and other investigators have been able to demonstrate a rising incidence of IDU-IE [23]. Unfortunately, due to little research in this area, the real extent of the problem remains to be determined. What has made it more difficult is the lack of specific WHO ICD-10 (World Health Organisation, International Statistical Classification of Disease 10) coding for IDU-IE [24].

Characteristically, we observe that people who inject drugs exhibit poor health-seeking behavior with a significant proportion presenting late [16]. It is not surprising, therefore, that in this study, >70% (17/23) were admitted to the ICU (intensive care unit). Of these, 88% (15/17) were ventilated as part of the management of severe infection and sepsis. The median ICU LOS was 4 days (IQR; 2 to 40) and the median hospital LOS was 40 days (IQR; 5 to 119). More than 70% had hospital LOS >4 weeks. In addition, because of the complex nature of these presentations, a significant amount of resource is automatically allocated to address specific needs [10, 25].

From 22 patient presentations (23 episodes) with a diagnosis of IDU-IE, the total cost of treatment for all the episodes at our centre was $1,628,359 AUD (median cost of $61363 per admission) (Table 1). In Australia, the cost of health care is funded by tax payers through Medicare services. This is a gross estimation based on DRG and WIES calculations [13, 14]. As an example of how DRG shapes funding, patient episode 6, LOS 5 days (DRG code A06B), is disproportionately higher than patient episode 5, LOS 30 days (DRG code F61Z) (Table 1). This can be attributed to the high-cost DRG starting with A. Patient 6 required a tracheostomy and ventilation >95 hours. In addition, they required multiple interventions (cardiac surgery, orthopaedic surgery, extra corporeal membrane oxygenation, and hemofiltration), and unfortunately, they died due to multiorgan failure [14].

The cost approximations derived from this study only represent a portion of the total cost incurred from IDU-related morbidity and mortality. In our estimations, we did not account for expenditure incurred from other comorbid conditions (e.g., toxic effects of drugs, complications from BBV (hepatitis C, hepatitis B, and HIV), and other bacterial infections other than IE), or loss of productivity due to illness in a cohort of youthful working-age adults [10, 25].

In conclusion, we show that infectious endocarditis, the result of injection drug use, results in a significant cost burden. Our study had a number of limitations: first, the lack of accurate ICD-1O coding for IDU-IE with a potential to miss cases; second, the study was done in a large regional cardiothoracic centre, so it may be possible that only severe cases were referred to our cardiothoracic centre, thereby skewing the conclusions; third, our cost estimates were based on a unique Medicare Australia and the state of Victoria DRG coding and WIES estimates for costing which may not be a generalisable technique of cost estimation. Lastly, we caution that the findings of this study may not be easily extrapolated given the small sample size and the retrospective nature of the study. Further studies are therefore required to accurately estimate the true economic burden of illicit drug use and associated infectious complications.

## Figures and Tables

**Table 1:** Cost per admission as derived from DRG (diagnosis related group) and WIES (weighted inlier equivalent separation) calculations.

Patient episode	Length of stay (days)	DRG (diagnosis related groups), category	Cost ($AUD) based on 2015/2016 WIES
1	27	F61Z, infective endocarditis (IE)	—
2	20	F61Z, Z63A, IE and other aftercare with catastrophic or severe complication or comorbidity (CC)	23,405
3	64	A06Z, IE, tracheostomy or ventilation >95 hrs	119,927
4	41	F03Z, cardiac valve procedure, CPB pump, invasive cardiac investigation	59,155
5	30	F61Z, IE	20,415
6	5	A06B, tracheostomy with ventilation >95 hours without catastrophic CC or trach/vent >95 hrs with catastrophic CC	30,559
7	34	F03A, cardiac valve proc with CPB pump with invasive cardiac investigation with catastrophic (cat) CC (complication or comorbidity)	68,670
8	52	F06A, coronary by-pass without invasive cardiac investigation with catastrophic or severe CC	74,336
9	26	F03A, cardiac valve proc CPB pump with invasive cardiac investigation with catastrophic complication or comorbidity	63,571
10	33	A06A, tracheostomy with ventilation >95 hours with catastrophic CC	168,361
11	68	F04A, cardiac valve proc with CPB pump without invasive cardiac investigation with catastrophic CC	95,953
12	47	A06A, tracheostomy with ventilation >95 hours with catastrophic CC	182,285
13	48	F04A, cardiac valve procedure with CPB pump without invasive cardiac investigation with catastrophic CC	66,592
14	20	F61A, infective endocarditis with catastrophic complication or comorbidity	30,097
15	24	F43Z, circulatory system diagnosis with non-invasive ventilation	17,327
16	49	E62A, I27A, respiratory infection/inflammation and soft tissues procedures with CC	30,435
17	1	F42C, IE	2806
18	39	E62A, respiratory infection and inflammation	23,170
19	119	A06A, tracheostomy with ventilation >95 hours and CC	266,357
20	45	A06A, tracheostomy with ventilation >95 hours and CC	171,841
21	43	F04A, F61A, infective endocarditis with CC	64,591
22	6	A06C, tracheostomy and ventilation	33,942
23	10	T64A IE and other infectious diseases and parasitic diseases with CC	14,564
Total			1,628,359.00
Median cost per admission			61363.00

IE, infective endocarditis, CC, complication or comorbidity.

**Table 2:** Characteristics of patients with IE-IDU.

	*n* (*n* = 23)	ICE	Heroin	Died	ICU admission	Valve surgery
		8	16	3	17	12
Male sex (%)	11 (47%)	6 (75%)	5 (33%)	1 (33%)	8 (47%)	6 (50%)
*S. aureus*	19 (83%)	6 (75%)	14 (88%)	3 (100%)	14 (82%)	8 (66%)
*Candida sp.*	1 (4%)	—	1 (6%)	—	—	1 (8%)
*Strep. sp.*	3 (13%)	2 (25%)	1 (6%)	—	3 (18%)	2 (17%)

ICE: methamphetamine; RSIE, right sided infective endocarditis; LSIE, left sided infective endocarditis; LSIE and RSIE, left and right sided Infective endocarditis.

## Data Availability

The clinical and demographic data supporting the findings of this study are restricted by the Barwon Health Institutional Review Board. The data are available from Dr Ohide Otome o.hide@hotmail.com for researchers who meet the criteria for access to confidential data.
